# A Feasible Machine Learning Approach to Improve Cognitive Screening in Multiple Sclerosis

**DOI:** 10.3390/biomedicines14102262

**Published:** 2026-10-06

**Authors:** Michelangelo Dini, Letizia Turchi, Giulia Gamberini, Alessandra Caporali, Giovanna Lucchini, Marta Tacchini, Luca Riccardo Chiveri, Mariaemma Rodegher, Letizia Leocani

**Affiliations:** 1 Faculty of Psychology, Vita-Salute San Raffaele University, 20132 Milan, Italy; dini.michelangelo@hsr.it (M.D.);; 2 Department of Neurorehabilitation Sciences, Casa Di Cura Igea, 20144 Milan, Italy; 3 Faculty of Medicine, Vita-Salute San Raffaele University, 20132 Milan, Italy

**Keywords:** multiple sclerosis, cognitive impairment, neuropsychological assessment, screening, machine learning, MoCA, BICAMS

## Abstract

**Background**: Cognitive impairment is a frequent manifestation of multiple sclerosis (MS). Although the Brief International Cognitive Assessment for Multiple Sclerosis (BICAMS) is the recommended screening battery, the Montreal Cognitive Assessment (MoCA) is more commonly used in routine clinical practice. The ability of published MoCA cutoffs to detect MS-specific cognitive impairment remains unclear. **Objective**: This study aimed to evaluate the diagnostic performance of published Italian MoCA cutoffs for MS-specific cognitive impairment and develop a machine learning (ML) pipeline to improve screening accuracy. **Methods**: We prospectively enrolled 222 people with MS who underwent both MoCA and BICAMS assessment. Cognitive impairment was defined as performance below the 5th percentile on at least one BICAMS test. The sensitivity, specificity, and balanced accuracy of four published Italian MoCA cutoffs were calculated. An elastic-net penalized logistic regression model incorporating age, education, and raw MoCA score was trained using nested five-fold cross-validation to predict cognitive impairment. We additionally evaluated the impact of disability and fatigue on cognitive impairment and model performance. **Results**: Cognitive impairment was identified in 62 (27.9%) participants. Raw MoCA scores were significantly lower in cognitively impaired participants than in cognitively preserved participants (22.68 ± 4.29 vs. 25.77 ± 3.67; *p* < 0.0001). Published MoCA cutoffs, however, showed extreme specificity (0.92–0.99) but below-chance sensitivity (0.10–0.44), yielding low balanced accuracy (0.54–0.68). The ML model achieved good discrimination (ROC-AUC = 0.787, 95% CI 0.717–0.852), with sensitivity = 0.69, specificity = 0.72, and balanced accuracy = 0.71. **Conclusions**: Published Italian MoCA cutoffs have insufficient sensitivity for screening MS-specific cognitive impairment. An ML-based scoring approach substantially improves sensitivity while maintaining acceptable specificity, offering a practical tool to enhance cognitive screening in MS.

## 1. Introduction

Cognitive impairment (CI) is one of the most frequent and disabling manifestations of multiple sclerosis (MS), affecting approximately 40–65% of patients during the disease course [1,2]. Cognitive deficits may emerge even in the early stages of the disease [3]. The cognitive domains most affected show substantial inter-individual variability, with deficits in information processing speed, memory, and executive functions [4]. Importantly, CI substantially impacts quality of life, occupational functioning, treatment adherence, and social autonomy, making early identification a major clinical priority [5].

Currently, the Brief International Cognitive Assessment for Multiple Sclerosis (BICAMS) is considered the gold-standard screening battery for CI in MS [6,7]. The BICAMS has demonstrated good psychometric properties and strong international validation [7,8]. However, administration and scoring require trained personnel and dedicated time, resources that are often unavailable outside specialized MS centres. Consequently, cognitive screening is still inconsistently performed in everyday neurological practice [9,10].

In contrast, the Montreal Cognitive Assessment (MoCA) is one of the most widely used cognitive screening tools in general clinical settings because of its brevity, ease of administration, and minimal resource requirements [11]. Although originally developed for screening mild cognitive impairment and dementia [11], several studies have suggested that the MoCA may also show good discriminant validity for detecting CI in MS populations [12,13]. These findings suggest that the MoCA may represent a practical alternative for preliminary cognitive screening in MS. Indeed, studies have also found that the MoCA is administered much more frequently than the BICAMS in everyday clinical practice, to screen for cognitive deficits in people with MS (pwMS) [9,10]. However, important limitations remain. Unlike the BICAMS, the MoCA was not designed to capture the characteristic cognitive profile of MS, which is often dominated by deficits in information processing speed and episodic memory rather than the cognitive syndromes typically observed in ageing and neurodegenerative disorders. As a result, the applicability of these normative thresholds to MS-related cognitive dysfunction remains uncertain [12].

This issue may be particularly relevant in clinical screening contexts. Cognitive changes in MS are often subtle and may emerge long before overt functional decline becomes apparent [14,15]. Therefore, these normative thresholds may not adequately identify clinically meaningful cognitive difficulties in people with MS, potentially limiting the usefulness of the MoCA as a stand-alone screening instrument in this population [12].

Recent studies have begun to explore machine learning (ML) approaches to improve cognitive evaluations in MS, for example, by combining a neuropsychological battery with ML-based feature selection to enhance diagnostic capacity [16] or by applying ML classifiers to multimodal neuroimaging data to detect subtle cognitive changes [17]. These approaches typically require specialized test batteries not yet available or unfeasible for routine clinical use (e.g., due to lack of validation in multiple languages and/or long administration times) or advanced diagnostic instrumentation and/or highly specialized clinical personnel unavailable outside specialized centres. To our knowledge, no prior study has applied an ML approach directly to data already collected during standard MoCA administration to correct for its known limitations in MS, without requiring any additional testing, imaging, or specialized personnel. In this context, ML can be implemented not to leverage large amounts of multimodal data but rather to provide an all-in-one tool that can improve the diagnostic accuracy of existing neuropsychological tests while adding no burden or minimal burden on clinical operations.

Therefore, the present study aimed to evaluate the ability of currently available Italian MoCA normative cutoffs to identify MS-specific cognitive impairment, using the BICAMS as the reference standard. In addition, we investigated whether an ML approach designed for everyday clinical practice could improve the identification of cognitive impairment relative to existing normative thresholds.

## 2. Materials and Methods

### 2.1. Study Population

A total of *N* = 222 consecutive pwMS were prospectively recruited from the inpatient neurorehabilitation service of Casa di Cura Igea (Milan, Italy) between January 2023 and March 2026. All participants underwent a standardized neuropsychological evaluation including both MoCA and BICAMS. Both neuropsychological batteries were administered in one single session, by expert neuropsychologists (G.G., A.C., and G.L.), as per routine clinical care protocol. The MoCA is a cognitive screening battery that includes brief tests of executive functions, attention, language, visuospatial abilities, orientation, and memory [11]. The BICAMS is the consensus gold-standard screening battery for MS-specific cognitive impairment and includes the Symbol–Digit Modalities Test (SDMT, assessing processing speed, attention, working memory, and visual search), the California Verbal Learning Test—2nd Edition (CVLT-II, assessing verbal memory), and the Brief Visuospatial Memory Test—Revised (BVMT-R, assessing visuospatial memory) [18].

To account for the potential impact of fatigue on cognitive performance [19], we also collected Modified Fatigue Impact Scale—21 items (MFIS-21) [20,21] data from routine clinical assessments, including total score and physical, cognitive, and psychosocial subscales.

Inclusion criteria were: (i) MS diagnosis, (ii) age ≥ 18 years, (iii) ability to undergo formal neuropsychological testing (i.e., absence of severe comprehension and/or vision and/or upper limb motor deficits that could limit the feasibility or validity of any test or subtest of either MoCA or BICAMS).

Written informed consent was obtained from all participants. All data were fully anonymized prior to analysis, and the study protocol was approved by the institutional ethics committee (protocol number: 1162_2022).

### 2.2. Definition of Cognitive Impairment

MoCA-defined cognitive impairment was defined based on four separate cutoffs derived from four published Italian MoCA validation studies. All selected cutoffs (Aiello et al. [22] = 18.58, Conti et al. [23] = 17.36, Dapor et al. [24] = 17.95, and Santangelo et al. [25] = 15.55) were applied after adjusting the raw total MoCA score for confounding effects of age and education, as per published scoring procedures, and corresponded to the 5th percentile of a normal distribution (−1.645 SD).

MS-specific cognitive impairment was defined based on the presence of at least one BICAMS test score <−1.645 standard deviations (SD) from the normative mean, based on published Italian normative data [26], to match the cutoff for cognitive impairment adopted by all aforementioned MoCA validation studies. There is currently no agreement as to a gold-standard approved cutoff for the BICAMS, although there is wide agreement regarding the use of cutoffs between −1.5 SD and −2 SD from the normative mean. We mirrored the threshold of the Italian normative MoCA cutoffs to enable fair comparisons of diagnostic accuracy. Indeed, given the null hypothesis of equal true diagnostic performance, using a higher cutoff for the BICAMS than for the MoCA (e.g., BICAMS = −1.5 SD, MoCA = −1.645 SD) would cause systematic bias in diagnostic performance. The choice to define cognitive impairment based on at least one test score below the cutoff was also informed by an Italian consensus, which recommends defining impairment based on impaired performance in 20–30% of performed tests [6,27]. Since the BICAMS includes three tests, impairment on one of them reflects impairment in 33% of the overall battery.

### 2.3. Statistical Analysis

All analyses were performed using Python 3.14 [28,29,30,31]. Results are displayed as mean ± standard deviation (SD) or median (1st–3rd quartile); the 95% confidence interval (95% CI) is provided where appropriate. All statistical tests were two-sided (α = 0.05).

First, we evaluated the known-group validity of the raw total MoCA score via an independent sample *t* test (grouping variable: BICAMS-defined cognitive impairment). Then, we evaluated the classification agreement between MoCA-defined and BICAMS-defined cognitive impairment descriptively (separately for each published cutoff) via 2 × 2 confusion matrices, which allow us to quantify true positives (TPs), false positives (FPs), true negatives (TNs), and false negatives (FNs). From these, we calculated sensitivity $\left(\frac{TP}{TP+FN}\right)$, specificity $\left(\frac{TN}{TN+FP}\right)$, and balanced accuracy $\left(\frac{sensitivity\;+\;specificity}{2}\right)$.

We then trained an elastic-net penalized logistic regression model, to predict MS-specific cognitive impairment. We adopted a machine learning framework to prioritize out-of-sample prediction accuracy over in-sample parameter estimation and interpretation. This approach reduces the risk of overfitting and ensures higher generalizability of results by effectively simulating predictive performance on new, unseen cases. To ensure robust estimation of model performance and minimize overfitting, we implemented nested cross-validation. The dataset was partitioned into five outer folds to estimate generalization performance, while an inner 5-fold cross-validation loop was used exclusively for hyperparameter tuning. Splitting was performed in a stratified way to ensure that the proportion of cognitively impaired/preserved participants was consistent across all train/test splits. The area under the receiver operating characteristic curve (ROC-AUC) was used as an evaluation metric for both inner and outer fold training. Polynomial expansion, standardization and the elastic-net model were combined in a single pipeline, which was refitted on the training data at every level of the nested cross-validation to preserve the integrity of the validation process. Model performance was pooled across outer folds to obtain an unbiased estimate of predictive accuracy and its 95% confidence interval (CI). The classification threshold for the deployed ML was selected as the mean of five outer-fold threshold values, which were calculated by maximizing Youden’s J index (sensitivity + specificity − 1) on outer-training out-of-fold predicted probabilities. Ties were resolved in favour of higher sensitivity, as the MoCA is primarily intended as a cognitive screening tool [11].

The predictors included age, years of education, and raw MoCA score, while the binary outcome variable was BICAMS-defined cognitive impairment status (impaired vs. not impaired). We deliberately limited the predictor set to these three variables based on the rationale that a deployed prediction pipeline requires complete data for every input feature. Including other predictors, such as the Expanded Disability Status Scale (EDSS), which is not always administered at every visit, could limit the usability of the predictive model in everyday clinical practice. Conversely, age and years of education can be easily collected during routine clinical visits (indeed they must be collected as part of standard MoCA administration in Italy), meaning that no additional data collection burden is imposed on healthcare practitioners even when the MoCA is the only assessment being performed at a given timepoint. The predictor set was expanded using second-degree polynomials (resulting in 9 total features: 3 linear, 3 quadratic, and 3 interactions), to capture potential non-linear associations and interaction effects between demographic variables and MoCA performance.

To enable a fairer comparison, we obtained 95% confidence intervals for the sensitivity, specificity, and balanced accuracy of each published cutoff and of the ML model via bootstrap resampling (2000 resamples, stratified by cognitive impairment status). We also formally compared the ML model’s classification against each published cutoff using McNemar’s exact test, stratified by ground-truth impairment status to separately test differences in sensitivity (among truly impaired patients) and specificity (among truly preserved patients), given the paired nature of the data. We additionally computed the paired difference in balanced accuracy between the ML model and each cutoff (using the same resampled patients for both, preserving the pairing). A two-sided bootstrap *p*-value was derived from the resulting distribution of differences.

The potential impact of fatigue on cognitive performance was investigated first by calculating Spearman’s correlation coefficients (*r*) between MFIS-21 scores and MoCA, SDMT, CVLT-II, and BVMT-R scores. The false discovery rate (16 total comparisons) was controlled using the Benjamini-Hochberg procedure [32].

The impact of fatigue on ML-based cognitive impairment prediction was investigated by fitting logistic regressions with MS-specific cognitive impairment as dependent variable and ML-based predicted probabilities and standardized MFIS-21 score as predictors (including the interaction term). Separate logistic regressions were fitted for MFIS-21 total score and the three subscales (physical, cognitive, psychosocial). The impact of disability severity (as measured by EDSS scores) was evaluated using the same methodology and standardized EDSS scores.

## 3. Results

The majority of study participants were female (60.8%) and had progressive MS, long disease duration and severe disability. MS-specific cognitive impairment was detected in 62 (27.9%) study participants, and 23 (10.4%) were impaired in at least two BICAMS tests. Table 1 reports detailed descriptive statistics of demographic, clinical, and cognitive features of the study sample.

**Figure 1:**
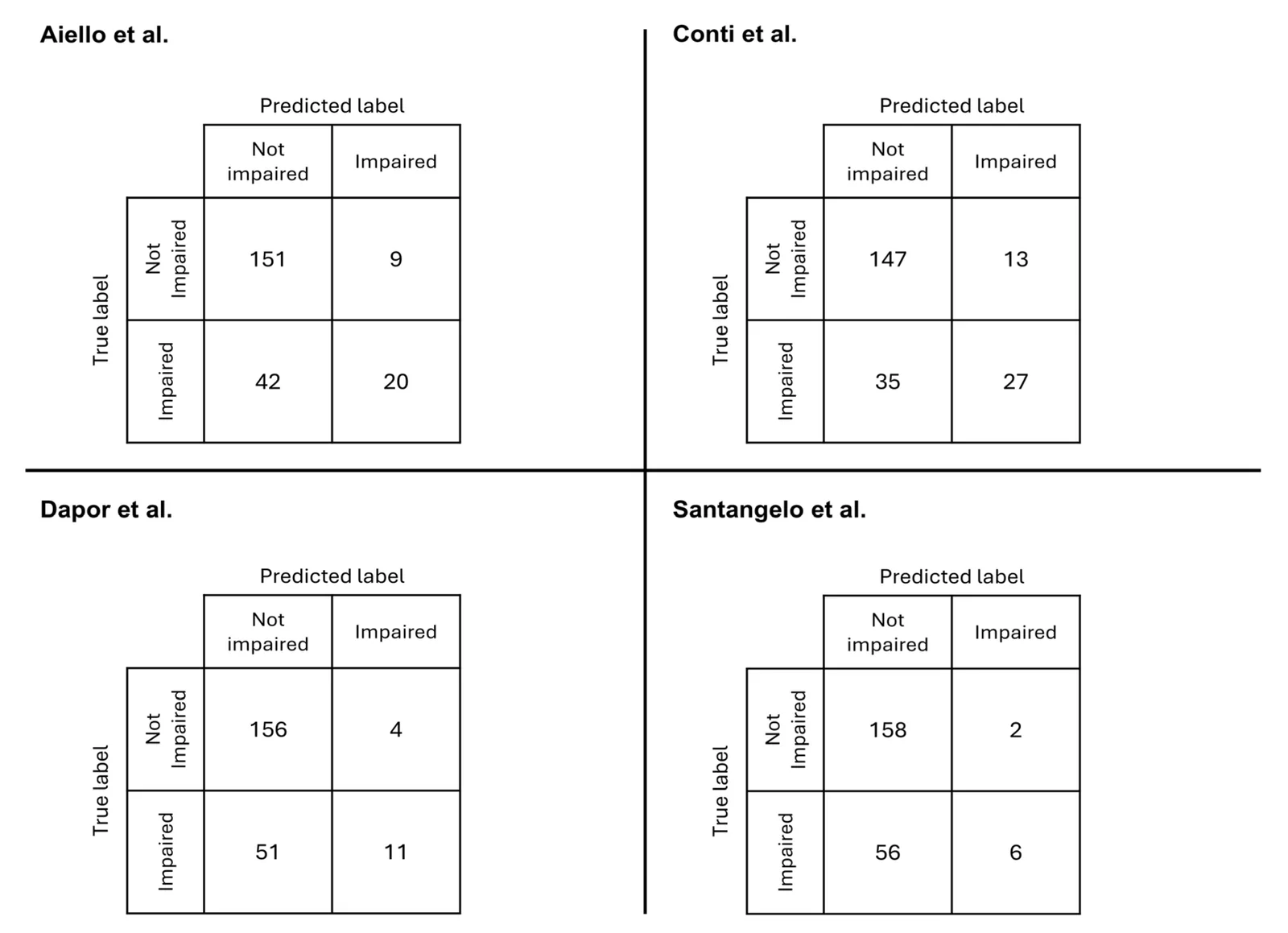
Classification performance of published Italian MoCA cutoffs. Confusion matrices comparing classification of cognitive impairment based on each of the four published Italian normative MoCA cutoffs (predicted label), relative to ground truth MS-specific cognitive impairment (true label). True positives = lower right quadrant, true negatives = upper left quadrant, false positives = upper right quadrant, false negatives = lower left quadrant.

Raw MoCA score was significantly lower in the cognitively impaired group (impaired vs. not impaired: 22.68 ± 4.29 vs. 25.77 ± 3.67, *p* < 0.0001), with a large effect size (Hedges’ *g* = 0.802). All four published MoCA cutoffs showed very high specificity (range = 0.92–0.99) but below-chance sensitivity (range = 0.10–0.44) and low balanced accuracy (range = 0.54–0.68) for MS-specific cognitive impairment. Detailed metrics are reported in Table 2 and Figure 1.

**Figure 2:**
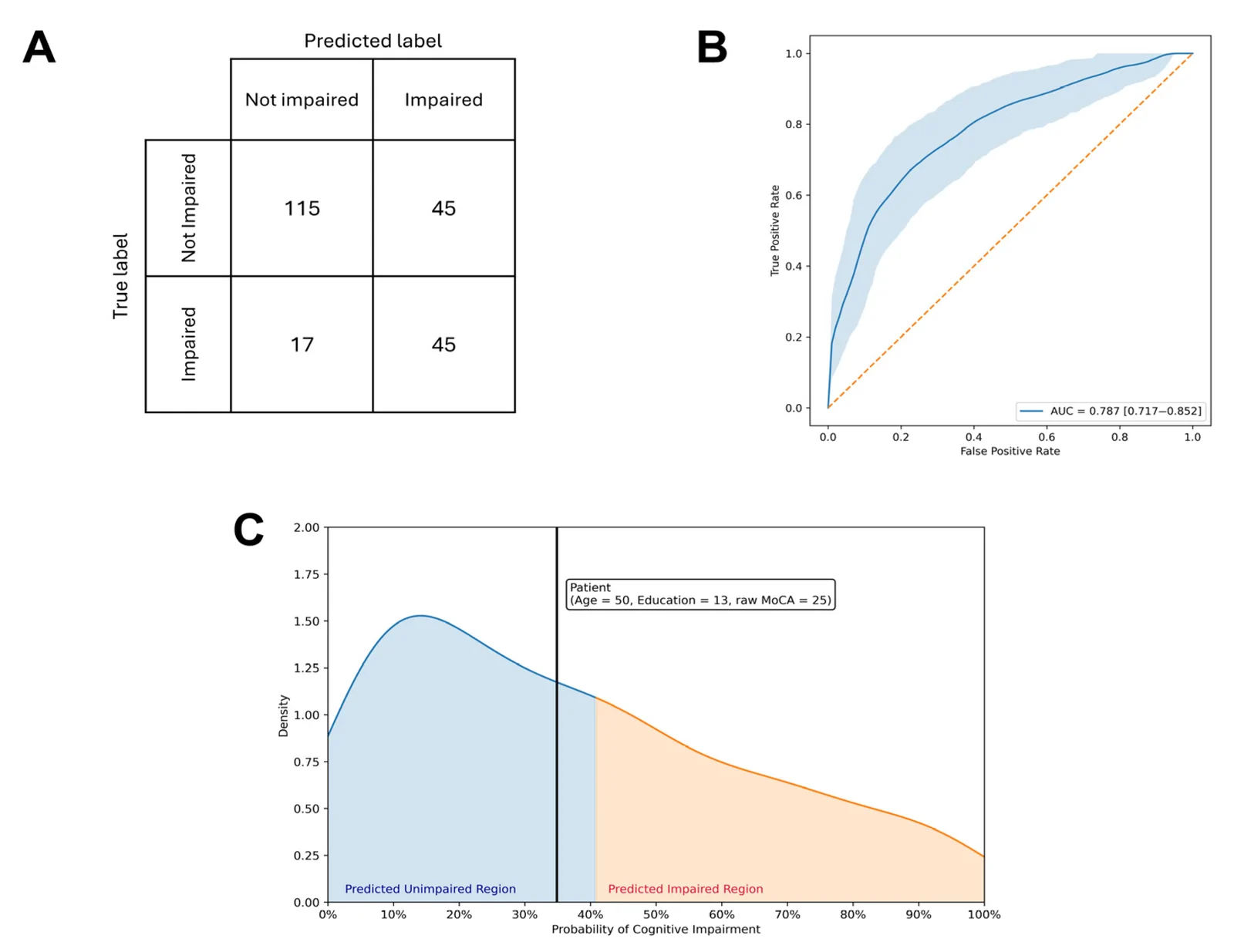
Performance of machine learning pipeline. (**A**) Confusion matrix comparing classification of cognitive impairment based on age, education, and raw MoCA score (predicted label), relative to ground truth MS-specific cognitive impairment (True label). (**B**) Receiver operating characteristic curve (solid blue line) with bootstrapped 95% confidence interval (light blue shaded area) to evaluate predictive performance across different decision thresholds. (**C**) Kernel density estimates distribution plot of predicted probabilities of cognitive impairment. The black solid vertical line represents the predicted probability of a hypothetical participant, while the decision threshold is represented as the separation between the two decision regions of the predicted probability distribution.

Concerning ML pipeline validation, the outer training and test sets comprised *N* = 177 and *N* = 45 observations per fold, respectively, with a consistent proportion of positive cases across splits (e.g., Fold 1: outer train = 49/177, 27.7%; outer test = 13/45, 28.9%). Across the five outer folds, the model achieved a mean ROC-AUC = 0.807 (95% CI = 0.729–0.885), mean sensitivity = 0.695 (SD = 0.155), mean specificity = 0.725 (SD = 0.064), and mean balanced accuracy = 0.710 (SD = 0.052), indicating good discriminative ability. An estimate of generalization performance based on pooled out-of-fold (OOF) predictions yielded a slightly lower but comparable ROC-AUC = 0.787 (95% bootstrapped CI= 0.717–0.852) (Figure 2B).

**Figure 3:**
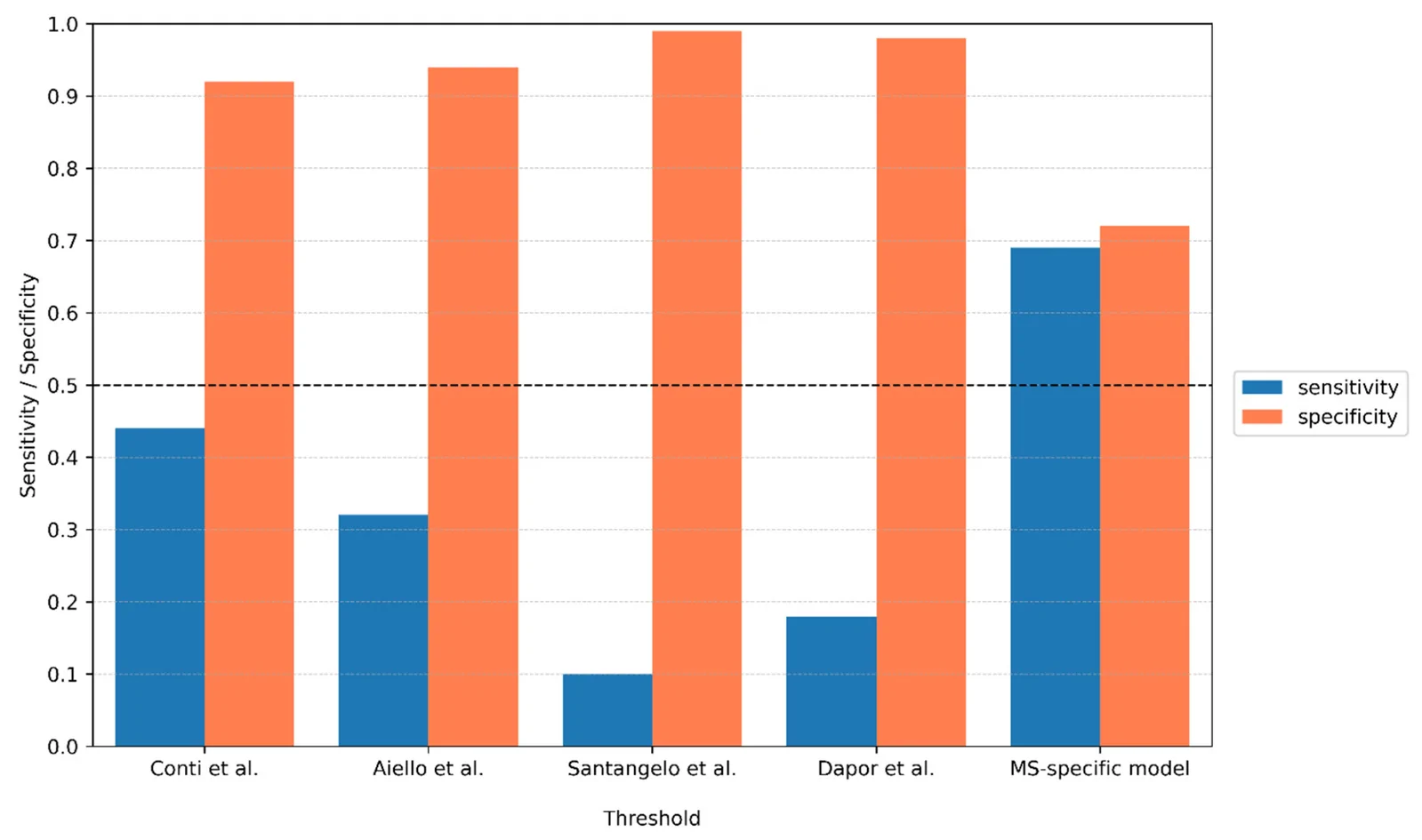
Comparison of cognitive impairment detection performance. Bar chart comparison of sensitivity (left bar) and specificity (right bar) of the four published MoCA cutoffs for the Italian population (Conti et al. [23], Aiello et al. [22], Santangelo et al. [25], and Dapor et al. [24]) and of the machine learning pipeline trained on our study sample of people with multiple sclerosis. The black horizontal dashed line represents 50/50 (random) chance.

For implementation of the final model, the mean outer-fold threshold = 0.407 was selected to avoid the optimistic bias that arises when a threshold is both selected and evaluated on the same set of out-of-fold predictions. Each of the five constituent thresholds was derived solely from outer-training out-of-fold predicted probabilities. Once applied to the pooled OOF sample, this cutoff yielded sensitivity = 0.726, specificity = 0.719, and balanced accuracy = 0.722, which are comparable values to those observed during validation. The full confusion matrix for this model and the predicted probability distribution are displayed in Figure 2A and Figure 2C, respectively. A comparison of prediction performance between the published Italian MoCA cutoffs and the ML pipeline is provided in Table 2 and Figure 3.

McNemar’s exact tests confirmed that the ML model’s sensitivity advantage over each cutoff was statistically significant (all *p* ≤ 0.0001), as was its specificity disadvantage relative to each cutoff (all *p* < 0.0001). Detailed results are reported in Table 2. For balanced accuracy, the ML model’s advantage was statistically significant against Dapor et al. (balanced accuracy difference = +0.133, *p* < 0.0001), Santangelo et al. (balanced accuracy difference = +0.167, *p* < 0.0001), and Aiello et al. (balanced accuracy difference = +0.077, *p* = 0.041) but not statistically significant against Conti et al. (balanced accuracy difference = +0.033, *p* = 0.353). It should be noted that the balanced accuracy of published cutoffs is pushed by the aforementioned very high specificity, as they present a large delta between specificity and sensitivity (delta range = 0.48–0.89). Balanced accuracy of the ML model, however, is a much more fitting estimate due to the small gap between sensitivity and specificity (delta = 0.03).

There were no significant correlations between cognitive performance (MoCA, SDMT, CVLT-II, and BVMT-R scores) and reported fatigue (MFIS-21 total score and all subscales). Detailed results are reported in  Supplementary Figure S1.

Fatigue did not affect ML-based predictions as evidenced by non-significant interaction terms between predicted probabilities and MFIS-21 total score (β = 0.035, *p* = 0.820), physical score (β = 0.014, *p* = 0.924), cognitive score (β = 0.091, *p* = 0.568), and psychosocial score (β = 0.073, *p* = 0.624). MFIS-21 total score (β = 0.431, *p* = 0.023) and MFIS-21 cognitive subscale (β = 0.685, *p* = 0.001) were significant independent predictors of cognitive impairment, whereas MFIS-21 physical (β = 0.115, *p* = 0.528) and psychosocial (β = 0.322, *p* = 0.088) subscales were not. Detailed results are reported in Supplementary Tables S1–S4.

EDSS score was also a significant independent predictor of cognitive impairment (β = 0.383, *p* = 0.048) but did not affect ML-based predictions (β = 0.018, *p* = 0.915). Detailed results are reported in Supplementary Table S5.

## 4. Discussion

We showed that, while raw MoCA score has discriminant power to distinguish pwMS with/without cognitive impairment, published Italian cutoffs demonstrate insufficient sensitivity, making the test unsuitable for cognitive screening in MS. This is a significant issue, considering the widespread diffusion and use of the MoCA for cognitive screening, especially in primary care settings. Indeed, low sensitivity leads to many false negatives, which lead to delayed referrals, late interventions, and ultimately a worse prognosis. Our study proposes a solution by providing clinicians with an ML-based MoCA scoring pipeline that improves sensitivity (from 44% to 69%) and maintains acceptable specificity (72%). This tool was designed to be easy to use in real-world clinical practice to ensure the broadest possible adoption across all levels of clinical care. To ensure usability at all levels of clinical care, we packaged the ML pipeline as a web app, freely available at https://github.com/michedini/Multiple-Sclerosis-MoCA-Predictor (accessed on 24 September 2026). The link provides an intuitive graphic interface that clinicians can use to obtain predictions of MS-specific cognitive impairment based on MoCA performance.

The observed difference in raw MoCA performance between cognitively impaired and cognitively preserved participants supports the construct validity of the test in MS. Participants classified as cognitively impaired according to the BICAMS scored, on average, more than three points lower than cognitively preserved individuals, with a large effect size. This finding is consistent with previous studies showing significant associations between MoCA performance and more-comprehensive neuropsychological measures in MS [12,13]. Although the MoCA was not specifically developed to detect the cognitive profile typically associated with MS, it is well suited to capture executive functioning and verbal memory deficits, which are frequent in MS [14]. Thus, even if other relevant domains such as processing speed and visuospatial memory are not covered by the MoCA, its total score appears capable of capturing clinically meaningful differences in overall cognitive functioning [14].

Despite this discriminative capacity at the group level, all four Italian normative cutoffs performed poorly in identifying individuals with MS-specific cognitive impairment. While specificity remained very high across thresholds, sensitivity was consistently below chance level, resulting in a substantial proportion of cognitively impaired individuals being misclassified as cognitively preserved. Consequently, balanced accuracy remained modest, suggesting that these thresholds may be overly conservative when applied to MS populations.

From a clinical perspective, this finding is particularly concerning. The primary aim of a screening instrument is to identify individuals who may require more-comprehensive diagnostic assessment rather than to establish a definitive diagnosis. Consequently, false-negative classifications may delay the recognition of cognitive impairment, postponing referral for neuropsychological assessment and limiting the timely implementation of appropriate management strategies and cognitive rehabilitation interventions [33]. Similar concerns have been raised by Ilardi et al. [34], who emphasized that normative studies should not only establish reference values in healthy populations but also evaluate the diagnostic performance of cognitive tests in specific clinical conditions. Notably, their study demonstrated that optimal MoCA cutoffs for detecting mild cognitive impairment and Alzheimer’s disease were substantially higher (approximately ranging between 21 and 23 points) than several thresholds currently proposed by Italian normative studies, highlighting the potential discrepancy between normative classification and clinical screening performance.

This observation is also broadly consistent with evidence from other countries. The original MoCA validation study by Nasreddine et al. [11] proposed a cutoff of 26/30 for the identification of cognitive impairment. Although subsequent studies in North American [35,36] and European countries [37,38] suggested that lower cutoffs may improve specificity without substantially compromising sensitivity, the recommended thresholds generally remained considerably higher than those from several Italian normative investigations. For example, systematic reviews and meta-analyses [39,40] have highlighted that optimal MoCA cutoffs often fall within the range of 23–24 points, depending on demographic characteristics and clinical context. As a result, Italian impairment thresholds are often considerably lower than those used internationally. While such thresholds may maximize specificity in healthy populations, they may be less suitable for detecting the relatively subtle and heterogeneous cognitive deficits typically observed in MS.

This discrepancy may reflect methodological as well as demographic factors. MoCA performance is known to be influenced by age, education, cultural background, linguistic characteristics, and geographical variability [36,40]. As highlighted by Carson et al. [40], the definition of an optimal cutoff cannot rely exclusively on normative considerations but should also account for the intended purpose of the assessment and the characteristics of the target population. Variability in sampling procedures, age distributions, educational attainment, and regional characteristics across normative studies may all contribute to differences in the resulting thresholds [34]. However, regardless of the specific reasons underlying these discrepancies, the present findings suggest that thresholds developed primarily to identify statistically abnormal performance in healthy individuals may not adequately address the clinical needs of MS populations.

Another important issue concerns the distinction between research and clinical objectives. In research settings, lower cutoffs associated with very high specificity may be desirable when cognitive impairment is used as an exclusion criterion and minimizing false-positive classifications is a priority. In routine clinical practice, however, the opposite situation often applies [34]. Screening instruments are typically expected to maximize sensitivity, thereby reducing the risk of missing individuals who may require further assessment. Within this framework, the low sensitivity observed for all Italian normative thresholds raises concerns regarding their suitability as standalone screening criteria in MS.

The ML pipeline improved the sensitivity of the MoCA to MS-specific cognitive impairment, while maintaining good specificity. Crucially, this was achieved by leveraging only raw MoCA score, age, and education. This is relevant for two main reasons. First, it ensures that inference on cognitive impairment is made based only on a widely available cognitive test [11] with supported validity in MS [41,42] and on easily collected variables that are known covariates for cognitive performance in the neuropsychological literature [11,18,43]. It must be stressed that sensitivity remained only acceptable, with one out of three pwMS with true cognitive impairment still being labelled as “not impaired”. It is possible that this reflects an inherent performance ceiling for the MoCA in MS, caused by the emphasis on domains that are not typically impaired in pwMS (e.g., language, orientation). However, the evidence regarding ample use of the MoCA for cognitive screening in MS (even when it is widely accepted that the BICAMS represents a better screening tool) suggests that tools to improve cognitive screening in routine clinical practice where the BICAMS may not be administered are sorely needed.

Fatigue (as self-reported via the MFIS-21) and disability severity (as measured via EDSS score) did not affect the predictions of the ML model. EDSS score, total MFIS-21 score and cognitive MFIS-21 score were, however, significant independent predictors of true cognitive impairment. This suggests that disability severity, fatigue and MoCA-detectable cognitive signal are at least partially distinct constructs. Some studies found significant correlations between fatigue and processing speed and learning [19]. Other studies, conversely, found no significant associations between fatigue and objective cognitive performance in pwMS [44], and a recent meta-analysis found disability severity to be a key determinant of cognitive performance in pwMS but no clear effect of fatigue [45]. Notably, this meta-analysis found that the effect of EDSS was moderate (correlation coefficients ranging between −0.50 and −0.34), independent of disease duration, and not necessarily related to confounding effects of sensory/motor deficits on neuropsychological test execution [45]. Further studies are needed, however, to disentangle the relative contribution of sensory and/or motor deficits to cognitive performance in pwMS, while the lack of a clear effect of fatigue on cognition may be attributable to methodological heterogeneity across studies. These include differences in sample characteristics (e.g., percentage of participants with RRMS or PPMS/SPMS, disease duration, disease severity) and assessment tools (e.g., cognitive tests, fatigue scales).

Including other predictors that are more indirectly correlated with cognitive performance in MS (e.g., EDSS and/or MFIS-21 score or other clinical variables; see, for example, [4]) may improve accuracy, but this also affects the construct validity of the prediction, reflecting broader disease severity (which in turn affects cognitive status too) and potentially different but interconnected physiological/psychological symptoms, rather than cognitive performance specifically. Our approach ensures feasibility in the broadest possible range of real-life clinical contexts, ranging from general practice to specialist centres. Indeed, for an ML model to be broadly deployable, the full set of predictor variables should be routinely available. Thus, while adding other clinical (e.g., EDSS score, disease duration) or radiological (e.g., brain MRI lesion load) predictors could likely improve classification accuracy, it would also reduce applicability in clinical settings in which such data may not be available.

An additional consideration is that the observed discriminative performance of the ML model may partly reflect overlap in the cognitive constructs assessed by the MoCA and the BICAMS. The MoCA includes measures of attention, executive functioning, and memory, which overlap with some of the domains assessed by the BICAMS, particularly verbal memory and aspects of attention and executive functioning. Consequently, the ROC-AUC should not be interpreted as representing discrimination against a completely independent cognitive construct. As discussed above, however, the MoCA also assesses domains such as orientation and language that are not directly represented in the BICAMS. Therefore, the observed AUC likely reflects both shared and non-shared aspects of cognitive functioning. The ML model should consequently be viewed as a tool for predicting BICAMS-defined cognitive impairment rather than as evidence of fully independent criterion validity.

As the MoCA allows for quantification of various sub-scores, reflecting more-specific cognitive domains (executive functions, attention, language, visuospatial abilities, orientation, memory), it could be argued that including these sub-scores as separate predictors may improve prediction accuracy and provide a more granular characterization of the cognitive impairment profile of pwMS. We did not perform this in our study, which was based on analysis of data collected as part of routine clinical care, in which only total MoCA score was recorded. Other studies have analysed MoCA sub-scores, to provide a more in-depth characterization of MoCA performance in pwMS. Podda et al. [4] identified four MS cognitive phenotypes based on MoCA sub-scores, showing that memory impairment was a common characteristic of all four phenotypes, while other domains showed distinct impairment patterns. Future studies may substitute the MoCA total score with the full set of sub-scores to predict MS-specific cognitive impairment. This could provide better accuracy by basing predictions on cognitive domains that are known to be most frequently affected in pwMS, like verbal and visuospatial memory [46]. However, one should always be mindful of how this impacts applicability of the model at scale and in real-world clinical practice. Indeed, scoring MoCA sub-scores is much more burdensome than total MoCA score, and the construct validity of the different sub-scores is not a subject of universal agreement in the literature [47,48]. Thus, MoCA sub-scores are unlikely to be widely available in clinical practice (as demonstrated by the lack of data in our sample), which may be especially true at primary care levels where screening is most crucial.

In addition to the abovementioned sensitivity improvement, an ML pipeline for predicting cognitive impairment has some notable advantages over conventional scoring approaches. First, it automatically and consistently performs all preprocessing (e.g., standardization, normalization) and scoring procedures internally, so that users only need to input the raw data to get a final prediction (impaired vs. not impaired) and its associated probability (e.g., 80% probability of belonging to the “impaired” class), which can provide further clinical decision support in edge cases. Conventional scoring, conversely, usually requires transformations to be calculated manually, as scoring software is not routinely made available alongside normative cutoffs. Indeed, to the best of our knowledge, no such MoCA scoring tools are available to clinicians in Italy. Therefore, raw score adjustments (which usually include square root- or log-based transformations) must be made manually based on equations published in the normative papers, which is time-consuming and could cause mistakes. Consequently, especially in primary care settings, raw score adjustment is commonly performed through conversion tables that group age and education in bins, thus introducing systematic bias upon score conversion. That is, the score of a male, 54 y.o. patient with 12 years of education and that of a male, 57 y.o. patient with 14 years of education would be both adjusted based on values for male patients with age = 55 and education = 13, leading to systematic over- and under-adjustment, respectively.

The limitations of this study include the predominance of people with progressive MS and severe disability in our sample, which may limit the generalizability of our model to people with RRMS and lower disability. More specifically, evidence points not only to a higher prevalence of cognitive impairment in progressive MS [14,49] but also to different qualitative impairment patterns, with progressive MS showing higher prevalence of attention/executive function deficits [46]. This could affect the diagnostic accuracy of the MoCA and the BICAMS in different ways, based on the relative importance of attention and/or executive function deficits in each battery. Importantly, this means that the diagnostic accuracy of the MoCA may be lower in patients with RRMS than in those with progressive MS. Our study was also based on a relatively large sample, but from a single centre. Although the ML approach partly solves this issue by focusing on out-of-sample validation, prediction accuracy may be lower in samples with completely different socio-demographic and clinical characteristics. It should be noted that this is a limitation shared by the traditional cutoff scoring approach in neuropsychology, in which the features and representativeness of the normative sample influence the accuracy of predictions. This is attested by the variability observed across the four published MoCA cutoffs for the Italian population, which was shown to cause systematic bias when comparing classifications based on different normative samples [22]. A further limitation concerns model stability. Although elastic-net regularization was used specifically to guard against overfitting, sensitivity varied more across the five outer cross-validation folds than specificity or balanced accuracy. Future efforts will be aimed at establishing multi-centric validation studies to ensure the maximum possible generalizability of predictions across the full range of cognitive impairment in MS, as well as across different countries.

## 5. Conclusions

While the BICAMS remains the consensus gold-standard screening battery for cognitive impairment in pwMS, generalist screening tests like the MoCA still see greater use in everyday clinical practice. We provided an easy-to-use tool, internally validated via nested cross-validation in a relatively large sample of pwMS. The tool aims to enhance the use of non-MS-specific cognitive screening tools in everyday clinical practice by increasing the sensitivity of the total MoCA score to MS-specific cognitive impairment. This could improve the current state of cognitive screening in MS, which in turn would allow earlier interventions and potentially a more favourable disease prognosis for a large proportion of pwMS.

## Figures and Tables

**Table 1:** Descriptive statistics.

Variable	N (%)	Mean ± SD or Median (1st–3rd Quartile)
Age	222 (100%)	54.38 ± 10.13
Education	222 (100%)	13 (12–17)
Sex	Males = 87 (39.2%) Females = 135 (60.8%)	-
MS type	RRMS = 14 (6.3%) SPMS = 150 (67.6%) PPMS = 58 (26.1%)	-
EDSS score	211 (95.0%)	6.5 (6.0–6.5)
Disease duration (years)	176 (79.3%)	15.93 ± 9.44
Cognitive impairment	Impaired = 62 (27.9%) Not impaired = 160 (72.1%)	-
MoCA score	Impaired (Aiello et al. [22]) = 29 (13.1%) Impaired (Conti et al. [23]) = 40 (18.0%) Impaired (Santangelo et al. [25]) = 8 (3.6%) Impaired (Dapor et al. [24]) = 15 (6.8%)	24.81 ± 4.02
SDMT score	Impaired (Goretti et al. [26]) = 37 (16.7%)	39.04 ± 12.67
CVLT-II score	Impaired (Goretti et al. [26]) = 28 (12.6%)	51.16 ± 14.06
BVMT-R score	Impaired (Goretti et al. [26]) = 28 (12.6%)	19.42 ± 9.28
MFIS-21 total score	211 (95.0%)	31 (19–46)
MFIS-21 physical score	211 (95.0%)	19 (12–26)
MFIS-21 cognitive score	211 (95.0%)	7 (2–15.5)
MFIS-21 psychosocial score	211 (95.0%)	4 (1–6)

Descriptive statistics of the study sample. For continuous variables, the “N (%)” column reports the count (%) of non-missing datapoints or the proportion of participants with impaired cognitive test performance. BVMT-R = Brief Visuospatial Memory Test—Revised, CVLT-II = California Verbal Learning Test—2nd Edition, EDSS = Expanded Disability Status Scale, MFIS-21 = Modified Fatigue Impact Scale—21 items, MoCA = Montreal Cognitive Assessment, PPMS = primary progressive MS, RRMS = relapsing-remitting MS, SDMT = Symbol–Digit Modalities Test, SPMS = secondary progressive MS.

**Table 2:** Predictive performance of published cutoffs and machine learning model.

Cutoff	Sensitivity (95% CI)	Specificity (95% CI)	Balanced Accuracy (95% CI)
Aiello et al. [22]	0.32 (0.21–0.44)	0.94 (0.91–0.98)	0.63 (0.57–0.70)
Conti et al. [23]	0.44 (0.32–0.55)	0.92 (0.88–0.96)	0.68 (0.61–0.74)
Dapor et al. [24]	0.18 (0.08–0.27)	0.98 (0.95–0.99)	0.58 (0.53–0.63)
Santangelo et al. [25]	0.10 (0.03–0.18)	0.99 (0.97–1.00)	0.54 (0.51–0.58)
Machine learning model	0.69 (0.58–0.81)	0.72 (0.66–0.79)	0.71 (0.64–0.78)

Comparison between published Italian normative cutoffs [22,23,24,25] and the machine learning pipeline in terms of sensitivity, specificity, and balanced accuracy for predictions of MS-specific cognitive impairment.

## Data Availability

The data presented in this study are available on request from the corresponding author pending approval from the data protection officer of Casa di Cura Igea S.p.A, Milano, Italy.

## Supplementary Materials

The following supporting information can be downloaded at https://www.mdpi.com/article/10.3390/biomedicines14102262/s1, Table S1. Sensitivity analysis including MFIS Total Score; Table S2. Sensitivity analysis including MFIS Physical Score; Table S3. Sensitivity analysis including MFIS Cognitive Score; Table S4. Sensitivity analysis including MFIS Psychosocial score; Table S5. Sensitivity analysis including disability severity (EDSS score); Figure S1. Correlations between cognitive performance and reported fatigue. Heatmap of correlation strengths between Modified Fatigue Impact Scale - 21 items (MFIS-21) and Montreal Cognitive Assessment (MoCA), Symbol-Digit Modalities Test (SDMT), California Verbal Learning Test (CVLT), and Brief Visuospatial Memory Test - Revised (BVMT). All correlations are not statistically significant (all *p* > 0.234).
