# Supplementary material for: A Feasible Machine Learning Approach to Improve Cognitive Screening in Multiple Sclerosis
Source: Biomedicines. 2026 Oct 6;14(10):2262. doi: 10.3390/biomedicines14102262 (PMC13701295; doi:10.3390/biomedicines14102262)
Supplement: Supplementary file 1 [file biomedicines-14-02262-s001.zip › biomedicines-4512045-supplementary.pdf]

**Supplementary Table S1. Sensitivity analysis including MFIS Total Score**

| Predictor | β | SE | z | *p* | 95% CI |
|---|---|---|---|---|---|
| Intercept | −0.666 | 0.188 | −3.543 | <0.001 | −1.034 to −0.298 |
| Model predicted log-odds | 0.871 | 0.146 | 5.956 | <0.001 | 0.584 to 1.157 |
| MFIS Tot (z-score) | 0.431 | 0.189 | 2.281 | 0.023 | 0.061 to 0.802 |
| Model prediction × MFIS | 0.035 | 0.154 | 0.227 | 0.820 | −0.266 to 0.336 |

Model fit: $N = 211$; log-likelihood = −95.520; McFadden pseudo-$R^2 = 0.224$; likelihood-ratio test $p < 0.001$.

Dependent variable = BICAMS-defined cognitive impairment (0 = No, 1 = Yes)

**Supplementary Table S2. Sensitivity analysis including MFIS Physical Score**

| Predictor | β | SE | z | *p* | 95% CI |
|---|---|---|---|---|---|
| Intercept | −0.664 | 0.180 | −3.696 | <0.001 | −1.016 to −0.312 |
| Model predicted log-odds | 0.814 | 0.139 | 5.849 | <0.001 | 0.541 to 1.087 |
| MFIS Physical (z-score) | 0.115 | 0.183 | 0.632 | 0.528 | −0.242 to 0.473 |
| Model prediction × MFIS | 0.014 | 0.146 | 0.095 | 0.924 | −0.273 to 0.301 |

Model fit: $N = 211$; log-likelihood = −98.131; McFadden pseudo-$R^2 = 0.203$; likelihood-ratio test $p < 0.001$.

Dependent variable = BICAMS-defined cognitive impairment (0 = No, 1 = Yes)

**Supplementary Table S3. Sensitivity analysis including MFIS Cognitive Score**

| Predictor | β | SE | z | *p* | 95% CI |
|---|---|---|---|---|---|
| Intercept | −0.659 | 0.197 | −3.353 | 0.001 | −1.044 to −0.274 |
| Model predicted log-odds | 0.932 | 0.153 | 6.087 | <0.001 | 0.632 to 1.232 |
| MFIS Cognitive (z-score) | 0.685 | 0.204 | 3.354 | 0.001 | 0.285 to 1.085 |
| Model prediction × MFIS | 0.091 | 0.159 | 0.571 | 0.568 | −0.221 to 0.403 |

Model fit: $N = 211$; log-likelihood = −91.609; McFadden pseudo-$R^2 = 0.256$; likelihood-ratio test $p < 0.001$.

Dependent variable = BICAMS-defined cognitive impairment (0 = No, 1 = Yes)

**Supplementary Table S4. Sensitivity analysis including MFIS Psychosocial score**

| Predictor | β | SE | z | *p* | 95% CI |
|---|---|---|---|---|---|
| Intercept | −0.628 | 0.187 | −3.367 | 0.001 | −0.994 to −0.263 |
| Model predicted log-odds | 0.859 | 0.147 | 5.864 | <0.001 | 0.572 to 1.147 |
| MFIS Psychosocial (z-score) | 0.322 | 0.189 | 1.704 | 0.088 | −0.048 to 0.693 |
| Model prediction × MFIS | 0.073 | 0.148 | 0.490 | 0.624 | −0.217 to 0.362 |

Model fit: $N = 211$; log-likelihood = −96.831; McFadden pseudo-$R^2 = 0.213$; likelihood-ratio test $p < 0.001$.

Dependent variable = BICAMS-defined cognitive impairment (0 = No, 1 = Yes)

**Supplementary Table S5. Sensitivity analysis including disability severity (EDSS score)**

| Predictor | β | SE | z | *p* | 95% CI |
|---|---|---|---|---|---|
| Intercept | −0.616 | 0.183 | −3.365 | 0.001 | −0.974 to −0.257 |
| Model predicted log-odds | 0.841 | 0.145 | 5.778 | <0.001 | 0.555 to 1.126 |
| EDSS (z-score) | 0.383 | 0.193 | 1.981 | 0.048 | 0.004 to 0.761 |
| Model prediction × EDSS | 0.018 | 0.164 | 0.106 | 0.915 | −0.305 to 0.340 |

Model fit: $N = 211$; log-likelihood = −96.545; McFadden pseudo-$R^2 = 0.228$; likelihood-ratio test $p < 0.001$.

Dependent variable = BICAMS-defined cognitive impairment (0 = No, 1 = Yes)

**Supplementary Figure 1. Correlations between cognitive performance and reported fatigue.**

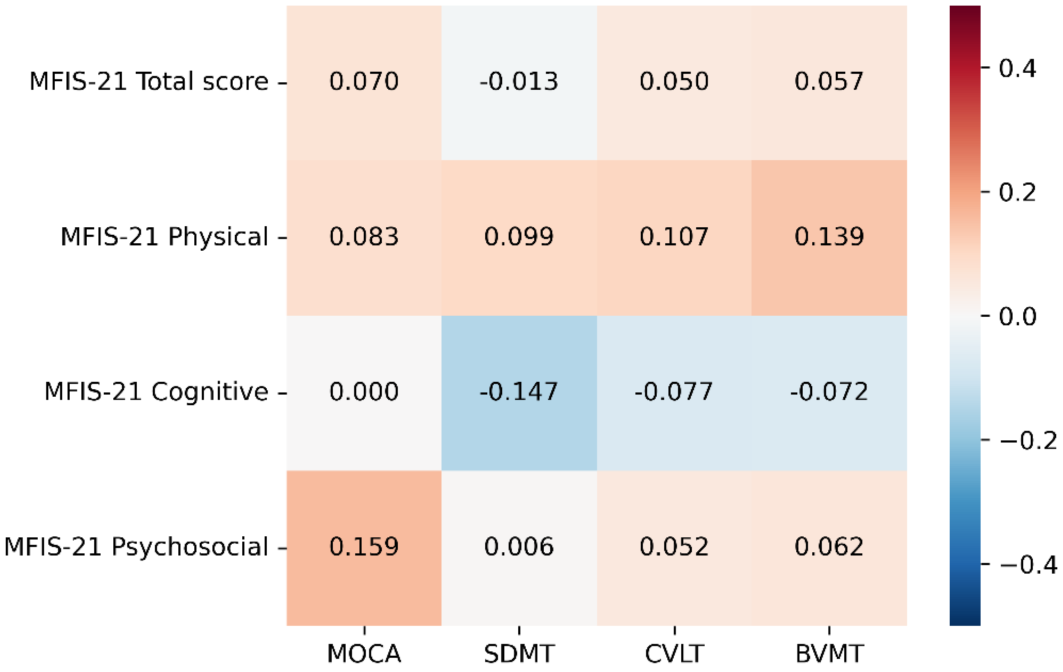


Heatmap of correlation strengths between Modified Fatigue Impact Scale - 21 items (MFIS-21) and Montreal Cognitive Assessment (MoCA), Symbol-Digit Modalities Test (SDMT), California Verbal Learning Test (CVLT), and Brief Visuospatial Memory Test - Revised (BVMT). All correlations are not statistically significant (all $p > 0.234$).
